# Differences in sarcopenia indices in elderly Japanese women and their relationships with obesity classified according to waist circumference, BMI, and body fat percentage

**DOI:** 10.1186/s40101-024-00370-7

**Published:** 2024-10-01

**Authors:** Chihiro Nishida, Motoyuki Iemitsu, Toshiyuki Kurihara, Keiko Kishigami, Motohiko Miyachi, Kiyoshi Sanada

**Affiliations:** 1 Faculty of Sport and Health Science, Ritsumeikan University, 1-1-1 Noji Higashi, Kusatsu, Shiga 525-8577 Japan; 2 Faculty of Science, Yamaguchi University, 3003 Yoshida, Yamaguchi, 753-8512 Japan; 3 Faculty of Sport Sciences, Waseda University, 2-579-15 Mikajima, Tokorozawa, Saitama 359-1192 Japan

**Keywords:** Sarcopenia, Obesity, Sarcopenic obesity, Waist circumference, BMI, Body fat percentage, Gait speed, Hand grip strength

## Abstract

**Background:**

Sarcopenic obesity (SO) is defined as a decrease in lean body mass and an increase in body fat mass (BFM) due to aging. Detecting SO in elderly women is important from the perspective of extending healthy life expectancy. While various indices of SO are currently used, there is no global consensus regarding diagnostic criteria for SO. This study aimed to examine the relationship between obesity indices (waist circumference (WC), body mass index (BMI), and body fat percentage (BFP)) and sarcopenia indices (total body muscle mass (TBM), appendicular lean mass (ALM), skeletal mass index (SMI)), and physical function (gait speed (GS), handgrip strength (HGS)).

**Methods:**

Subjects were 170 community-dwelling healthy elderly women aged 65–79 years (mean: 72.7 ± 5.78 years) who underwent measurements for WC, BMI, and BFP. A WC of ≥ 90cm was defined as the obese group, BMI was determined as weight (kg) divided by height squared (m^2^) and a cutoff of ≥ 25 kg/m^2^ was used to define the obesity group. BFM was measured using the bioelectrical impedance analysis (BIA) method and BFP was calculated from body weight and a cutoff of ≥ 30% was used to define the obesity group. TBM and ALM (kg) were measured using the BIA method, ALM (kg) was corrected for height (m^2^) to obtain SMI (kg/m^2^). Physical function was assessed by GS and HGS, which were measured by the 5-m walk test and a digital grip strength meter, respectively.

**Results:**

When obesity was assessed using BMI, WC and BFP, obese individuals had higher TBM, ALM and SMI, and lower GS among the sarcopenia indicators. HGS did not differ significantly between the non-obese and obese groups.

**Conclusion:**

Our findings suggest HGS is thought to reflect muscle strength without being affected by obesity indices, suggesting that it may be useful in detecting possible sarcopenia in obese individuals.

## Background

“Sarcopenia” is a condition related to the age-related decline of physical function and is defined by the European Working Group on Sarcopenia in Older People (EWGOSP, 2010) as “a syndrome characterized by progressive and generalized loss of skeletal muscle mass and strength with risk of adverse outcomes including physical disability, reduced quality of life, and death” [1]. Studies on sarcopenia suggest that the condition is associated with an increased risk of physical disability [2], development of osteoporosis [3], and falls [4, 5], as well as an increased risk of requiring long-term care. Furthermore, associations with metabolic syndrome [6] and risk of total mortality [7] have been noted.

In reference to the European Working Group on Sarcopenia in Older People (EWGSOP2, 2019) [8], the current diagnostic algorithm [9] for diagnosing sarcopenia of the Asian Working Group for Sarcopenia (AWGS, 2019) for Asians uses handgrip strength (HGS) to assess muscle strength, bioelectrical impedance analysis (BIA) or dual energy X-ray absorptiometry (DXA) to measure skeletal muscle mass, and gait speed (GS) to assess physical function. People with sarcopenia have a combination of low skeletal muscle mass and low muscle strength, or low skeletal muscle mass and low physical function. Those with low values for all three of these measures have severe sarcopenia.

Obesity is defined as “a state of excessive accumulation of fat cells” [10] and is widely recognized to increase the risk of cerebrovascular disease [11], diabetes [12], and cancer [13]. Tchkonia et al. concluded that the impact of body fat mass (BFM) on health in post-retirement elderly people is a chronically positive energy balance, which may accelerate the accumulation of excess adipose tissue and the development of age-related diseases [14]. Studies investigating the effects of obesity on aging have shown that it promotes cognitive decline [15], and that age-related changes in body fat distribution and metabolism may be an important factor in the vicious cycle that accelerates the aging process and the onset of age-related diseases [16]. Body mass index (BMI) is used worldwide as an indicator of obesity and is the primary criterion in the diagnostic flowchart for obesity. In addition to a BMI ≥ 25 kg/m^2^, obesity is diagnosed if a patient has obesity-related health problems or if the accumulation of visceral fat, which tends to increase health problems, exceeds the reference value [10]. In particular, visceral fat area is designated as an essential item for the determination of metabolic syndrome, and waist circumference (WC) is measured when screening for metabolic syndrome. Visceral fat accumulation is measured by computed tomography [17], and a visceral fat area accumulation of ≥ 100 cm^2^ is suspected when WC is ≥ 85 cm for men and ≥ 90 cm for women. Body fat percentage (BFP) analyzed from body composition by DXA or BIA is used as an indicator to determine the tendency for obesity in clinical settings. While BFP has not been adopted as a direct criterion for obesity, it is a powerful measure for assessing health status because excessive accumulation of BFM is known to have detrimental consequences on health.

Herber et al. defined sarcopenic obesity (SO) as a state of decreased lean body mass and increased BFM with aging [18]. Previous studies have shown SO to be closely related to age-related loss of bone mass, decreased basal metabolic rate, and increased BFP [19]. Higher blood pressure is associated with obesity, however sarcopenia with obesity is at a higher risk than sarcopenia alone [20], as well as a higher mortality risk [21]. SO, as defined by WC, increases all-cause mortality, but no equivalent trend has been observed for BMI or BFP, and the possibility that different obesity indices have different effects on mortality risk has been investigated [22]. Anja et al. reported a 1.5-fold increase in the hazard ratio of mortality risk in cases with high fat mass and low fat free mass (FFM), while the risk of death was reduced by 30% in the case of high FFM and low BFM. From these results, the authors concluded that prevention of excessive fat accumulation and maintenance of muscle mass in the elderly are key to preventing increased mortality [23]. The prevalence of SO is reportedly significantly higher in women, particular in elderly women, than in men [24, 25]. In addition, elderly women have a higher risk of joint disorders (knee osteoarthritis) than men [26], suggesting that SO in elderly women is more likely to interfere with their daily lives than in men.

Many previous studies on SO have used various indices as criteria for sarcopenia and obesity, and there is currently no global consensus on diagnostic criteria for SO [27]. The EWGOSP 2010 [1], EWGSOP 2019 [8] and AWGS [9] are diagnostic criteria for sarcopenia, but it has been reported that high BFP overestimates muscle mass when muscle mass is measured using conventional sarcopenia indicators [28–30]. Thus, it is currently unclear whether sarcopenia assessment methods are appropriate for obese individuals and whether sarcopenia indicators are valid for obese individuals.

Therefore, clarifying the relationship between sarcopenia indices and obesity indices in SO is important from the perspective of providing more accurate assessments. Elderly women are more affected by SO [25], however the relationship between these indices have not been studied in this population. Therefore, the present study aimed to examine the relationship between obesity indices (WC, BMI, and BFP) and sarcopenia indices (total body muscle mass (TBM), appendicular lean mass (ALM), skeletal mass index (SMI), as well as physical function (GS and HGS)), in elderly women.

## Methods

### Study subjects

Subjects were 170 community-dwelling healthy elderly women aged 65 to 79 years (mean 72.7 ± 5.78 years) recruited by advertisements in newspapers, magazines, and at lectures and other meetings for the public, as well as through e-mail and phone calls. After providing written and verbal explanations of the study’s purpose, details regarding measurements, and possible disadvantages of participating in the study at a preliminary briefing session, subjects who consented to participate were selected for the study. Exclusion criteria were: 1) secondary obesity due to adrenal gland disease, 2) heart disease or abnormalities in electrocardiograms, 3) serious liver dysfunction or cirrhosis, 4) pregnancy or suspected pregnancy, 5) undergoing orthopedic surgery or having exercise restrictions, and 6) considered by the principal investigator as not being appropriate to participate in the study. The present study was approved by the Biwako-Kusatsu Campus Bioethics Review Committee at Ritsumeikan University (approval number: BKC-LSMH-2021–072).

### Measurements, criteria, and classification of obesity

Three indices were used to determine obesity: WC, BMI, and BFP. WC was measured with a tape measure at the umbilical level while standing. A WC of ≥ 90 cm was defined as the obese group. BMI was determined as weight (kg) divided by height squared (m^2^), and a cutoff of ≥ 25 kg/m^2^ was used to define the obesity group. BFM was measured by the BIA method (Inner Scan 50 V RD-804L, TANITA, Tokyo, Japan). BFP was calculated based on body weight and a cutoff of ≥ 30% was used to define the obesity group.

### Measurement items for sarcopenia

TBM was measured by the BIA method (InnerScan50V, RD-804L, TANITA, Tokyo, Japan). ALM (kg) was corrected for height (m^2^) to obtain SMI (kg/m^2^) [1, 8, 9, 31]. GS and HGS were used to assess physical function [9]. GS was measured with a 5-m walking test. In the 5-m walking test, the subject starts walking 3 m before the start point, the measurement begins when the foot crosses the start point, and the time until both feet cross the 5-m end point is measured. HGS was measured twice on both sides (alternating) in the standing position.

### Statistical analysis

Results are expressed as mean ± standard deviation for each measurement. The normality of the data was tested by the Shapiro–Wilk test. The t-test was used to compare means between groups when a normal distribution was confirmed, and the Mann–Whitney test was used to compare means between groups when the distribution was not normal. Correlation coefficients between obesity indices and sarcopenia indices were calculated using the Pearson product-moment correlation coefficient (for normally-distributed indices) and Spearman’s rank correlation coefficients. *P* < 0.05 was considered statistically significant. SPSS Statistics Ver 29 (IBM) was used for statistical analyses.

## Results

### Subject characteristics

Table 1 shows the physical characteristics of the 170 subjects, including the three obesity indices (WC, BMI, and BFP) according to obesity status (i.e., obese or non-obese). The numbers of subjects in the obese groups when defined by a WC ≥ 90, BMI ≥ 25 kg/m^2^, and BFP ≥ 30% were 50, 39, and 105, respectively. There were no significant differences in age and height among the obese groups. Weight, WC, BMI, and BFP were significantly higher in the obese groups than in the non-obese groups.

**Table 1:** Subject characteristics. Subjects were classified into non-obese and obese groups according to three obesity indices

	**All Subjects (*n* = 170)**			**WC**							**BMI**							**BFP**						
				**N (*n* = 120)**			**O (*n* = 50)**				**N (*n* = 131)**			**O (*n* = 39)**				**N (*n* = 65)**			**O (*n* = 105)**			
**Measurements**	**Mean**		**SD**	**Mean**		**SD**	**Mean**		**SD**	***p***	**Mean**		**SD**	**Mean**		**SD**	***p***	**Mean**		**SD**	**Mean**		**SD**	***p***
**Age (years)**	72.7	±	5.8	72.8	±	5.8	72.5	±	5.8	*n.s*	72.9	±	6.0	72.0	±	5.1	*n.s*	72.7	±	6.3	72.7	±	5.5	*n.s*
**Height (cm)**	153.2	±	6.1	153.1	±	5.7	153.2	±	7.1	*n.s*	153.0	±	5.7	153.5	±	7.3	*n.s*	153.5	±	5.9	152.9	±	6.2	*n.s*
**Weight (kg)**	53.5	±	9.4	49.7	±	5.5	62.6	±	10.6	^**^	49.7	±	5.3	66.2	±	9.1	^**^	46.3	±	4.4	57.9	±	8.9	^**^
**WC (cm)**	84.0	±	10.1	79.0	±	6.8	96.1	±	5.1	^**^	80.4	±	8.0	96.2	±	5.9	^**^	75.8	±	6.8	89.1	±	8.3	^**^
**BMI (kg/m**^**2**^**)**	22.8	±	3.6	21.2	±	2.3	26.6	±	3.5	^**^	21.2	±	2.0	28.0	±	2.8	^**^	19.7	±	1.5	24.7	±	3.2	^**^
**BFP (%)**	32.0	±	7.2	29.0	±	5.5	39.0	±	5.7	^**^	29.1	±	5.1	41.6	±	4.2	^**^	24.8	±	3.4	36.4	±	5.1	^**^
**TBM (kg)**	34.0	±	4.0	33.2	±	3.1	35.9	±	5.0	^**^	33.2	±	3.1	36.6	±	5.3	^**^	33.1	±	2.7	34.5	±	4.5	^**^
**ALM (kg)**	15.3	±	2.2	14.9	±	1.5	16.5	±	3.1	^**^	14.9	±	1.5	16.9	±	3.3	^**^	14.7	±	1.6	15.7	±	2.5	^**^
**SMI (kg/m**^**2**^**)**	6.5	±	0.6	6.3	±	0.4	7.0	±	0.9	^**^	6.3	±	0.4	7.1	±	0.9	^**^	6.2	±	0.4	6.7	±	0.7	^**^
**GS (m/s)**	1.4	±	0.2	1.4	±	0.2	1.4	±	0.2	^*^	1.4	±	0.2	1.3	±	0.2	^**^	1.5	±	0.2	1.4	±	0.2	^**^
**HGS (kg)**	22.6	±	4.2	22.9	±	4.0	22.0	±	4.5	*n.s*	22.5	±	4.0	23.0	±	4.8	*n.s*	22.7	±	4.2	22.6	±	4.2	*n.s*

Classified into non-obesity and obesity groups based on three obesity indices. n.s, not significant

*WC* Waist circumference, *BMI* Body mass index, *BFP* Body fat percentage, *TBM* Total body muscle mass, *ALM* Appendicular lean mass, *SMI* Skeletal muscle mass index, *GS* Gait speed, *HGS* Hand grip strength

^*^p < 0.05, ^**^p < *0.01*

### Differences in sarcopenia indices between obese and non-obese groups

**Fig. 1:**
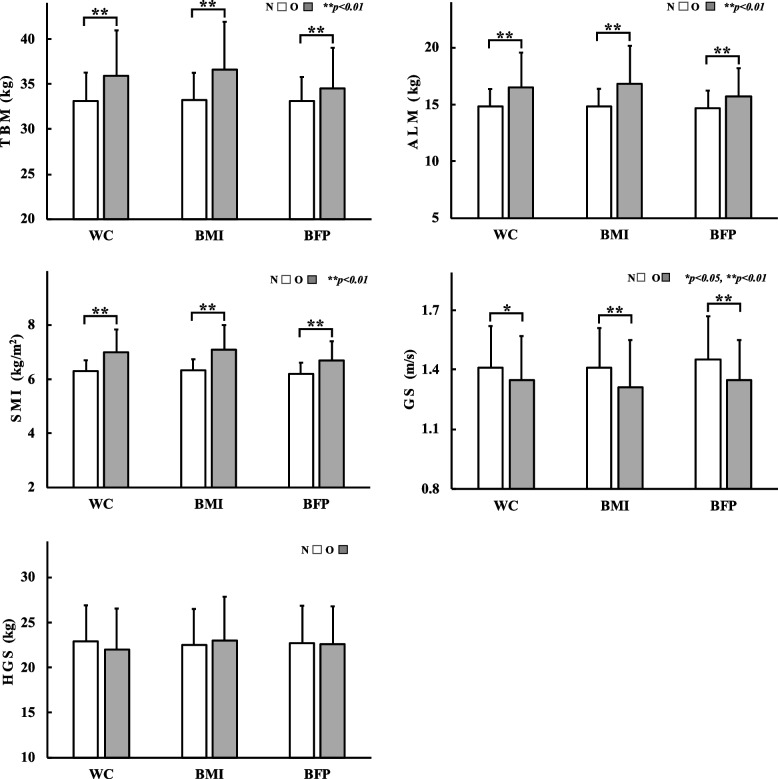
Relationships between obesity indices and sarcopenia indices for non-obese and obese groups. Subjects were classified into non-obese and obese groups according to the three obesity indices and subjected to comparisons of sarcopenia indices. Open square: non-obese group, closed square: obese group. WC, waist circumference; BMI, body mass index; BFP, body fat percentage; TBM, total body muscle mass; ALM, appendicular lean mass; SMI, skeletal muscle mass index; GS, gait speed; HGS, hand grip strength. *p < 0.05, **p < 0.01

When both groups were classified based on WC, BMI, or BFP (Fig. 1), the obese groups had significantly higher TBM, ALM, and SMI than the non-obese groups (*p* < 0.01). In terms of physical function (Fig. 1), the obese groups had significantly lower values for GS than the non-obese groups (classified on WC = *p* < 0.05, classified on BMI, BFP = *p* < 0.01). There were no significant differences in HGS between the obese and non-obese groups for any of the obesity indices.

### Correlations between obesity indices and sarcopenia indices

A correlation matrix of the measured indices is provided in Table 2. The three obesity indices (WC, BMI, and BFP) were positively correlated with TBM, ALM, and SMI (*p* < 0.01), while all obesity indices were negatively correlated with GS (*p* < 0.01). HGS was not significantly correlated with any of the obesity indices, while TBM, ALM, SMI and GS were all significantly, positively correlated with HGS (*p* < 0.01). The relationship between age and height was significantly negative (*p* < 0.01). Body weight was significantly, positively correlated with obesity indices (*p* < 0.01), as well as TBM, ALM, and SMI (*p* < 0.01).

**Table 2:** Correlation matrix of measured indices. Correlation coefficients among all assessed measures

		**Age (years)**	**Height (cm)**	**Weight (kg)**	**Obesity Indices**			**Muscle Mass Indices**			**Physical Function**
					**WC (cm)**	**BMI (kg/m**^**2**^**)**	**BFP (%)**	**TBM (kg)**	**ALM (kg)**	**SMI (kg/m**^**2**^**)**	**GS (m/s)**
**Height (cm)**		-0.374^**^									
**Weight (kg)**		-0.160^*^	0.312^**^								
**Obesity indices**	**WC (cm)**	0.031	0.059	0.784^**^							
	**BMI (kg/m**^**2**^**)**	0.03	-0.164^*^	0.853^**^	0.856^**^						
	**BFP (%)**	0.059	-0.115	0.827^**^	0.810^**^	0.936^**^					
**Muscle mass indices**	**TBM (kg)**	-0.378^**^	0.769^**^	0.682^**^	0.352^**^	0.318^**^	0.207^**^				
	**ALM (kg)**	-0.311^**^	0.711^**^	0.660^**^	0.354^**^	0.327^**^	0.230^**^	0.917^**^			
	**SMI (kg/m**^**2**^**)**	-0.124	0.117	0.628^**^	0.522^**^	0.618^**^	0.423^**^	0.593^**^	0.736^**^		
**Physical function**	**GS (m/s)**	-0.334^**^	0.351^**^	-0.114	-0.203^**^	-0.271^**^	-0.307^**^	0.185^*^	0.143	-0.039	
	**HGS (kg)**	-0.160^*^	0.442^**^	0.219^**^	-0.029	-0.029	-0.084	0.482^**^	0.434^**^	0.206^**^	0.209^**^

Correlation coefficients among all measures

*WC* Waist circumference, *BMI* Body mass index, *BFP* Body fat percentage, *TBM* Total body muscle mass, *ALM* Appendicular lean mass, *SMI* Skeletal muscle mass index, *GS* Gait speed, *HGS* Hand grip strength

^*^p < 0.05, ^**^p < *0.01*

## Discussion

The present study examined relationships between three obesity indices (WC, BMI, and BFP) and muscle mass indices (TBM, ALM, and SMI), as well as physical function (GS and HGS), in elderly women to determine which indices should be considered for observing SO. To our knowledge, this is the first report on the relationship between obesity indices and sarcopenia indices in elderly women. The main findings of the present study were (1) the obese groups had significantly higher TBM, ALM, and SMI than the non-obese groups; (2) GS was significantly lower in the obese groups than in the non-obese groups; (3) HGS did not significantly differ between the obese and non-obese groups. Based on these findings, it is suggested that 'obesity' should be considered when classifying sarcopenia, particularly in muscle mass and GS.

### Muscle mass (TBM, ALM, and SMI)

Muscle mass decreases with age, with 75-year-olds losing muscle mass at a rate of 0.64–0.7% per year in women and 0.8–0.98% in men [32]. The loss of skeletal muscle mass may also worsen Activities of Daily Living (ADL) and Instrumental Activities of Daily Living (IADL) in obese individuals [33], and thus the maintenance of bone-muscle mass is an important factor for the independent living of elderly people. However, the absolute maximum muscle strength of obese subjects is reportedly greater than that of non-obese subjects, and weight gain due to obesity acts as a chronic overload stimulus to antigravity muscles such as the quadriceps and calves, resulting in increased muscle mass [28]. In the present study, there were significant, positive correlation between body weight and TBM, ALM, and SMI (Table 2), suggesting that the physiological response of skeletal muscle to weight gain is reflected in the high values for all three muscle mass indices.

### Relationship between sarcopenia indices and age

TBM and ALM, which are sarcopenia indices, were negatively correlated with each other and age. However, there was no correlation between TBM, age, and SMI (Table 2). To obtain SMI [1, 8, 9], ALM is corrected by height squared, and the lower the height, the higher the SMI. In women, the rapid decrease in female hormone secretion associated with menopause affects bone metabolism, resulting in a shortening of height [34]. This may result in a high SMI value but may also be offset by a decrease in muscle mass due to aging. Therefore, age is an important factor to consider when evaluating muscle mass indices.

### Physical function

A) Gait speed (GS)

Mobility by walking is reduced in elderly people aged ≥ 65 years [35]. Moreover, being an elderly woman is a risk factor for the development of motor dysfunction in the knees and hip joint [36]. Obesity, when accompanied by muscle weakness, negatively impacts physical function [37], and the presence of obesity exacerbates GS [38]. GS of elderly people is an indicator that reflects health and functional ability [39], and maintaining it is an important factor in daily life. Subjects of the present study were healthy elderly women aged 65 to 79 years, and their GS ranged from 1.32 to 1.45 m/s, which does not meet the threshold criteria for sarcopenia [9]. It was thus assumed that most of our subjects did not have serious issues with physical activity. However, there was a negative correlation between aging and GS (Table 2), GS values were significantly lower in the obese group for all obesity indices (Fig. 1) and there was a negative correlation between obesity indices and GS (*p* < 0.01). This suggests that as the tendency of obesity increases in older age, there may be an excess accumulation of fat and a decrease in the quality of skeletal muscle, leading to a decrease in GS. The detrimental effects of obesity, such as infiltration of adipocytes into tissues, increased mild systemic inflammation, and loss of function [40, 41], may hinder the ability to exert adequate muscle strength for walking. In the present study, a positive correlation was found between body weight and TBM, ALM, and SMI, suggesting that weight gain increases muscle mass. This would have favorable effects on physical function [29], but the change in muscle mass does not necessarily reflect good physical function. GS has clinical significance as an indicator of physical ability and is suggested to be highly useful in identifying the presence or absence of functional impairment. However, it has also been reported that knee osteoarthritis, which is frequently observed in obese people, may affect the measurements [42]. Thus, the validity of using GS to diagnose sarcopenia in obese people must be more carefully examined.

B) Hand grip strength (HGS)

HGS has been widely used as a physical fitness measure and indicator of muscle strength in algorithms for determining sarcopenia [1, 8, 9]. HGS is also adopted as one of the diagnostic criteria in the SO consensus statement [43]. HGS varies according to the strength of finger and forearm muscles, which support essential activities in daily life, but as HGS and muscle mass decrease with aging, physical function deteriorates, and quality of life decreases. Progressive loss of HGS and muscle mass can also affect the risk of mortality [44, 45]. Thus, HGS can be viewed not only as a reflection of muscle strength and muscle mass, but also as a predictor of independent functional status and risk of mortality. Studies examining sarcopenia indices in elderly women have reported a significant positive correlation between muscle mass and HGS [46, 47], and the results of the present study similarly showed a positive correlation between muscle mass indices and HGS (TBM; *r* = 0.48, ALM; *r* = 0.43, SMI; *r* = 0.20, *p* < 0.01). In our subjects, HGS tended to decrease slightly with increasing age (*r* = -0.16, *p* < 0.05), but there was no significant difference among groups classified by the three obesity indices (Fig. 1) and no relationship with the obesity indices either (Table 2). In other words, HGS can reflect muscle mass without being affected by obesity status (i.e., regardless of whether obesity status is classified according to WC, BMI, or BFP). Therefore, it is a useful indicator for detecting the possibility of sarcopenia in obese individuals.

### Limitations

This study has several limitations. First, few of the subjects were judged to be sarcopenic when using the AWGS2019 criteria. Thus, while the tendency of muscle mass to increase with obesity was confirmed, we could not confirm that the relationship was statistically significant since only a few subjects had sarcopenia. Second, given the cross-sectional design of the study, the results captured a temporary phenomenon, and variations in obesity and skeletal muscle indices according to physical activity status remain unknown. In the future, more precise relationships between obesity and sarcopenia indices should be examined in longitudinal studies to allow more accurate indices for diagnosing sarcopenia in obese people to be determined.

## Conclusion

When obesity was assessed using BMI, WC and BFP, obese individuals had higher values for muscle mass and lower values for physical function among the sarcopenia indicators. However, the HGS was considered to reflect muscle strength without the influence of obesity indices, suggesting that it may be useful for detecting possible sarcopenia in obese individuals.

## Data Availability

The datasets used and/or analyzed during the current study are available from the corresponding author on reasonable request.

## Abbreviations

EWGOSP: The European Working Group on Sarcopenia in Older People
AWGS: The Asian Working Group for Sarcopenia
HGS: Handgrip strength
BIA: Bioelectrical impedance analysis
DXA: Dual energy X-ray absorptiometry
GS: Gait speed
BFM: Body fat mass
BMI: Body mass index
WC: Waist circumference
BFP: Body fat percentage
SO: Sarcopenic obesity
TBM: Total body muscle mass
ALM: Appendicular lean mass
SMI: Skeletal mass index
ADL: Activities of daily living
IADL: Instrumental activities of daily living
